# Sponge-supported cultures of primary head and neck tumors for an optimized preclinical model

**DOI:** 10.18632/oncotarget.25244

**Published:** 2018-05-18

**Authors:** Amy J.C. Dohmen, Joyce Sanders, Sander Canisius, Ekaterina S. Jordanova, Else A. Aalbersberg, Michiel W.M. van den Brekel, Jacques Neefjes, Charlotte L. Zuur

**Affiliations:** ^1^ Department of Head and Neck Oncology and Surgery, Antoni van Leeuwenhoek, Amsterdam, The Netherlands; ^2^ Department of Pathology, the Netherlands Cancer Institute - Antoni van Leeuwenhoek, Amsterdam, The Netherlands; ^3^ Department of Computational Cancer Biology, the Netherlands Cancer Institute - Antoni van Leeuwenhoek, Amsterdam, The Netherlands; ^4^ Center for Gynecological Oncology Amsterdam, VUmc, Amsterdam, The Netherlands; ^5^ Department of Oral and Maxillofacial Surgery, Academic Medical Center, University of Amsterdam, Amsterdam, The Netherlands; ^6^ Division of Cell Biology, the Netherlands Cancer Institute, Amsterdam, The Netherlands; ^7^ Department of Nuclear Medicine, the Netherlands Cancer Institute, Amsterdam, The Netherlands; ^8^ Department of Chemical Immunology, Leiden University Medical Center, Leiden, The Netherlands

**Keywords:** head and neck cancer, squamous cell carcinoma, primary tissue, cell culture, sponge gel supported histoculture

## Abstract

Treatment of advanced head and neck cancer is associated with low survival, high toxicity and a widely divergent individual response. The sponge-gel-supported histoculture model was previously developed to serve as a preclinical model for predicting individual treatment responses. We aimed to optimize the sponge-gel-supported histoculture model and provide more insight in cell specific behaviour by evaluating the tumor and its microenvironment using immunohistochemistry. We collected fresh tumor biopsies from 72 untreated patients and cultured them for 7 days. Biopsies from 57 patients (79%) were successfully cultured and 1451 tumor fragments (95.4%) were evaluated. Fragments were scored for percentage of tumor, tumor viability and proliferation, EGF-receptor expression and presence of T-cells and macrophages. Median tumor percentage increased from 53% at day 0 to 80% at day 7. Viability and proliferation decreased after 7 days, from 90% to 30% and from 30% to 10%, respectively. Addition of EGF, folic acid and hydrocortisone can lead to improved viability and proliferation, however this was not systematically observed. No patient subgroup could be identified with higher culture success rates. Immune cells were still present at day 7, illustrating that the tumor microenvironment is sustained. EGF supplementation did not increase viability and proliferation in patients overexpressing EGF-Receptor.

## INTRODUCTION

Seventy percent of head and neck squamous cell carcinoma (HNSCC) patients present with advanced stage disease. A vast majority of these patients is treated with surgery and/or high-dose cisplatin chemoradiotherapy (CCRT) or cetuximab based bioradiation. Despite these intensive treatment modalities, clinical outcome is characterized by a relatively low overall 5-year survival of 35–63% [1–3]. Furthermore, CCRT is associated with substantial toxicity, namely 89% of patients receiving CCRT for grade III and IV HNSCC, endured grade 3 or worse toxicity (CTCAEv3.0), compared to 52% of patients treated with single modality radiation [4]. A limited absolute overall survival benefit of 6.5% at 5 years for HNSCC patients treated with CCRT compared to RT alone is observed [5]. The choice between different strategies is mainly based on patient comorbidity, age and doctor preferences. Consequently, there is a strong need for a predictive test to select the optimal treatment. A pretreatment method could be a short-term culture model assessing *in vitro* response to different modalities. Ultimately, patients would then undergo individualized treatment regimens based on the *in vitro* tumor response.

Tumor culture assays have the potential to mimic the *in vivo* sensitivity, especially when the microenvironment and the heterogeneity of the tumor is maintained. A recent review summarized all preclinical models in HNSCC [6]. Our group performed a systematic review on primary HNSCC culture models and their ability to predict clinical response. We found that the most successful culture rates and best clinical correlations are obtained with the sponge-gel-supported histoculture, used as the histoculture drug response assay (HDRA) [7]. Leighton *et al.* developed this technique in 1951 in an effort to resemble a patient's tumor more accurately [8]. The technique preserves the 3D histological structure by using tumor fragments instead of cell lines. Furthermore, the sponge-gel-supported histoculture does not require additional enzymatic digestion, thus maintaining cell-cell interactions within the tumor tissue [8, 9]. This short-term assay hinders clonal evolution of tumor cell (sub)populations [10–13] and senescence [14, 15]. All cells, benign and malignant, are co-cultured together, supported by a sponge that allows for the formation of cell clusters with identifiable and distinctive tissue patterns. These are prerequisites to arrive at a preclinical culture model comparable to the *in vivo* tumor environment [8]. The group of Hoffman further developed this assay, in gastric and colorectal cancer, for clinical response applications [16, 17]. Robbins *et al*., however, were the first to test the HDRA technique on HNSCC tissue in 1994 [18]. Later, the HDRA model was adopted by several authors for preclinical chemosensitivity correlations in patients with head and neck cancer [7, 19–22].

Overall culture success rates of HNSCC with HDRA, are quite high; ranging from 88% to 100% [18, 19, 21, 22] with a culture duration varying from 2 to 11 days [18–21]. The main cause of culture failure is bacterial contamination. Looking at the correlation between *in vitro* chemosensitivity and clinical outcome in these studies, positive predictive values of 69% to 90% and negative predictive values of 50% to 100% were reported [18–21]. Interestingly, one study found improved predictive values by excluding patients that received adjuvant radiotherapy [19]. Despite these promising results, overall, the preclinical model did not allow individual clinical decision making and was therefore not taken into routine clinical practice.

To improve the HNSSC histoculture system, several aspects should be taken into account. Firstly, literature has reported that preclinical chemoresponses and radiation responses are dependent on the response of stromal cells surrounding the malignant cells. These studies indicated that chemosensitivity tests should be corrected for stromal cell content since they are more resistant for cytostatic drugs and radiation [23–25]. Secondly, the abundance of immune cells in the tumor microenvironment (TME) has not been evaluated in previous reports. Extracellular matrix, endothelial, stromal and infiltrating immune cells make up the bulk of the tumor environment and continuously interacts with cancer cells to sustain tumor progression and therapy resistance [26]. The TME affects treatment response and the prognosis of patients. An increased number of immune cells has been shown to correlate to an increased disease-free and overall survival [27, 28]. Thirdly, mainly fetal calf serum has been added to the medium in HDRAs of HNSCC [19–22], which could provide or deplete essential factors for healthy maintenance of the tumors in this culture system. The predictive value of the HDRA system for HNSCC may be improved by adding growth factors and other medium supplements sustaining viability of the cancer and stromal cells. Finally, so far, the HDRA assay in HNSCC has been performed with a metabolic cell viability read-out (MTT or tritiated thymidine incorporation). Using a metabolic read-out, one cannot differentiate between the various cells types present in the tissue.

With our research, we aim to evaluate the short-term sponge-gel-supported tumor histoculture for its abundance, viability and proliferation of malignant cells and surrounding stromal and immune cells using immunohistochemistry. In addition, we aim to test various supplements in the culture medium to support an optimal *in vitro* growth of HNSCC fragments. With these adaptations, we aim to optimize the histoculture for its potential use as an individual preclinical model to select the best individualized treatment regimens for HNSCC patients.

## RESULTS

### Patient, tumor and histoculture characteristics

Biopsies of 72 patients were taken under routine general anaesthesia and transported to our laboratory. After microscopic assessment of the fragments, we excluded 2 patients in which >50% of the fragments were contaminated with bacteria and fungi (70% and 86%) and 3 patients with >50% of the fragments containing mostly benign cells (67%, and two times 100%). Consequently, 93% of patient biopsies were successfully taken into culture. Furthermore, 6 patients were excluded in which a reliable day 0 statistical calculation was not possible since less than 3 fragments survived the procedure. Four patients with less than 3 fragments at day 7 for our control measurement, standard ‘RPMI’ medium, were also excluded. In total, 57 of 72 patients (79%) were included for analysis. For further details on patient and tumor characteristics, see Table 1. Of the 24 patients with oropharyngeal tumors tested, 16 tumors were HPV negative and 8 tumors were HPV positive. Fragments were fixated at day 5 for 2 patients and at day 8 for one patient, while all other patient samples were fixed at day 7. For readability, we will further refer to 7 days for all patients.

**Table 1:** Patient and tumor characteristics

Characteristics	Number of patients (*n* = 57)
**Gender**	
Male	36
Female	21
**Age**	
Age (years, median)	64
Range (years)	45–86
**Operating room**	
EUA^*^	38
Surgical resection	19
**Anatomical site**	
Oral cavity	16
Oropharynx	24
Hypopharynx	7
Larynx	10
**T-stage**	
T1/T2	27
T3/T4	30
**N-stage**	
N0	19
N1	5
N2	31
N3	2
**Stage**	
I/II	11
III/IV	46

^*^Examination under anaesthesia.

From the biopsies of 57 patients, we cultured 1451 tumor fragments in total. After microscopic assessment, 104 single fragments (7.2%) were of benign origin (gland or muscle tissue) and therefore excluded from further analysis. From the 1451 tumor fragments we excluded 35 fragments (2.4%) due to bacteria or fungi contamination and 32 fragments (2.2%) due to technical issues (tissue had disintegrated in culture, no tissue in cassette after the tissue processor machine, no tissue found in the paraffin block). Fragments taken from the hypopharynx site had the lowest successful culture efficiency, namely 83.2%, due to a high bacteria or fungi contamination rate of 13.8%. The total evaluability rate of the included 1451 fragments was 95.4% (see Table 2).

**Table 2:** Overview of tumor fragments per tumor site

	Number of patients	Number of tumor fragments	Contamination	Technical problem	Total succes
**Oral cavity**	16 (28.1%)	429 (29.6%)	2 (0.5%)	8 (1.9%)	419 (97.7%)
**Oropharynx**	24 (42.1%)	633 (43.6%)	4 (0.6%)	15 (2.4%)	614 (97.0%)
**Hypopharynx**	7 (12.3%)	167 (11.5%)	23 (13.8%)	5 (3.0%)	139 (83.2%)
**Larynx**	10 (17.5%)	222 (15.3%)	6 (2.7%)	4 (1.8%)	212 (95.5%)
**Total**	**57**	**1451**	**35 (2.4%)**	**32 (2.2%)**	**1384 (95.4%)**

### Culture efficacy in view of state, site and tumor proportion

Tumor viability and proliferation at day 7 did not relate to tumor stage or tumor site of origin, see Table 3. Also, we wondered whether a high percentage of cancer cells (raw median ≥70%) in the tissue sample at day 0 would benefit the culture efficacy. However, tumor viability and proliferation during culturing did not relate to the abundance of cancer cells at the start of culture, see Table 3. With ≥70% tumor cells at day 0 a median of 42% viability and 33% proliferation was seen, compared to respectively 30% and 25% with tissue with <70% tumor cells at day 0. As seen in Table 3, the results in the various samples vary widely and no significant differences could be extracted.

**Table 3:** Culture efficacy in view of state, site and tumor proportion

Variable	% Viability		% Proliferation		# of patients	Statistics
	Day 7	Range	Day 7	Range		
**Stage I**	10	5–80	35	1–50	*n* = 2	Viability *p = 0.263*^†^ Proliferation *p = 0.881*^†^
**Stage II**	42	0–300	29	0–167	*n* = 9	
**Stage III**	25	11–89	25	0–100	*n* = 6	
**Stage IV**	36	0–150	29	0–160	*n* = 40	
**Oral cavity**	27	0–95	33	0–160	*n* = 16	Viability *p = 0.051*^†^ Proliferation *p = 0.272*^†^
**Oropharynx**	31	0–300	25	0–156	*n* = 24	
**Hypopharynx**	47	0–100	22	0–133	*n* = 7	
**Larynx**	44	0–400	33	0–167	*n* = 10	
**≥70% Tumor day 0**	42	0–150	33	0–167	*n* = 18	Viability p = 0.559^*^ Proliferation p = 0.053^*^
**<70% Tumor day 0**	30	0–400	25	0–160	*n* = 39	

Median normalized percentages of viability and proliferation at day 7 for RPMI fragments, and its range (min – max), in view of stage of disease, tumor site and abundance of cancer cells present at day 0. (^†^Kruskal–Wallis test; ^*^Mann–Whitney *U* test.).

### Culture efficacy with RPMI condition

**Figure 1:**
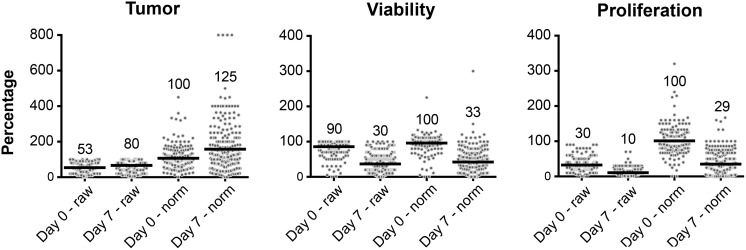
Culture efficacy data from all single tumor fragments (dots) at day 0 and day 7 cultured with standard RPMI Depicted are the raw and normalized values for each tumor fragment. Values shown in the graph correspond to the horizontal bar which depicts the median from all included fragments.

Using the standard ‘RPMI’ culture condition, the median tumor percentage increased from 53% at day 0 to 80% at day 7. The median viability of these cancer cells decreased from 90% at day 0 to 30% at day 7. The proliferation rate of the viable cancer cells also decreased, from 30% at day 0 to 10% at day 7. When comparing the normalized values of day 0 and 7 the same trend was observed (Figure 1).

### Optimization conditions

**Figure 2:**
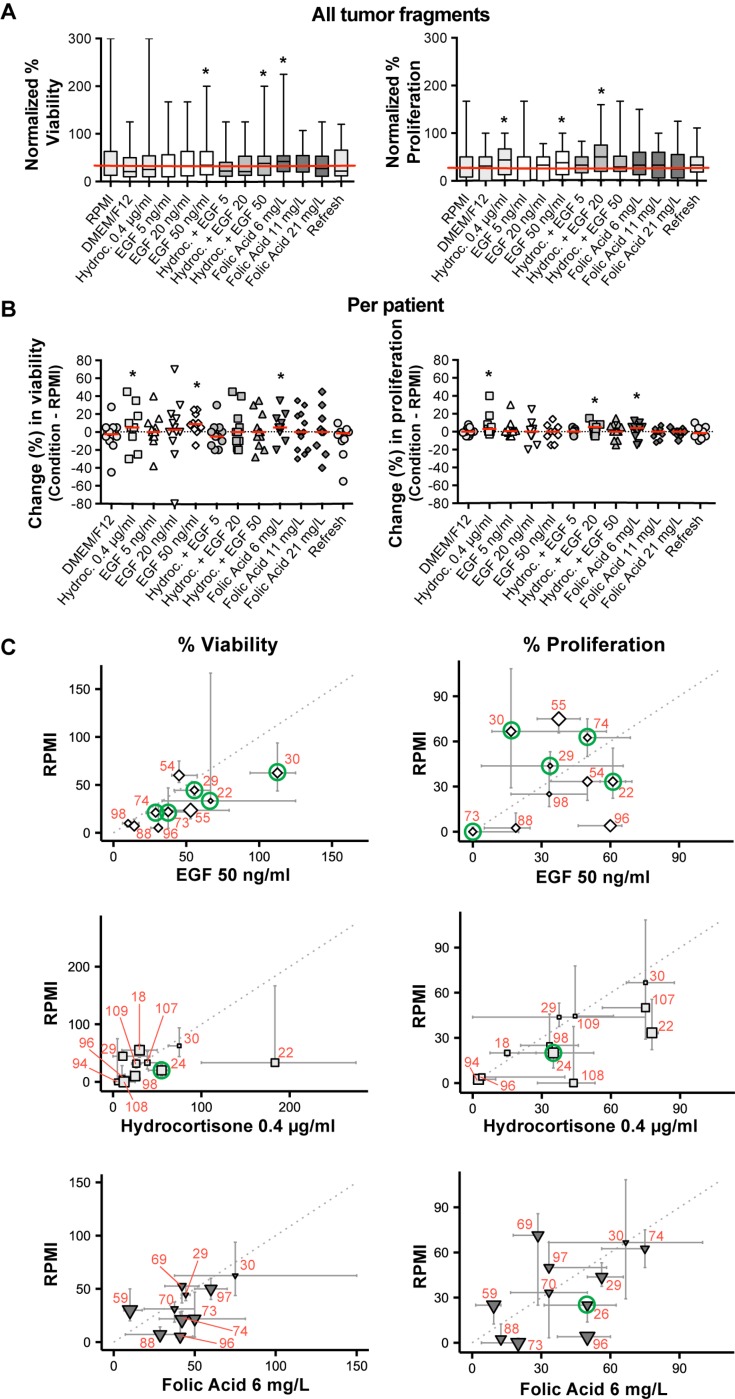
The effect of the culture optimization conditions on tumor viability and proliferation in comparison to standard RPMI, at day 7 (**A**) Boxplot of normalized viability and proliferation (median and range) of all tumor fragments cultured with the various optimization conditions. The horizontal red line delineates the median value for the standard RPMI. (^*^Best conditions.) (**B**) Scatter plot of raw median viability and proliferation percentages per optimization condition when compared to standard RPMI, depicted per individual patient. A single data point represents the difference between the median individual percentage of an optimization condition and the standard RPMI. The red bar indicates the median of all these single data points within that specific condition. (^*^Best conditions.) (**C**) Scatter plot of normalized data per patient for the three best selected optimization conditions (EGF 50 ng/ml ◊, Hydrocortisone 0.4 μg/ml □ and Folic Acid 6 mg/L ▼) versus standard RPMI. One symbol resembles the median of all fragments per individual patient; error bars around the symbols range from the first to the third quartiles. The size of the symbol is inversely proportional to the *p*-value of a two-sided Mann–Whitney *U* test comparing the RPMI and the tested optimization condition within one patient. The green circles indicate the selected samples for EGFR and immune cell IHC. For comparison between figures and tables, red numbers indicate individual patients. (Mind the axes that vary between the graphs.)

To optimize the histoculture efficacy in terms of tumor viability and proliferation, we tested various optimization conditions and compared the results to the standard ‘RPMI’ (Figure 2). In Figure 2A normalized percentages of viability and proliferation are shown for all tumor fragments taken together (not per patient), cultured with a specific supplement versus standard RPMI. RPMI reached a median viability of 33% at day 7. EGF 50 ng/ml (34%), hydrocortisone + EGF 50 ng/ml (38%) and folic acid 6 mg/L (42%) increased the median viability of the cultured fragments in comparison to RPMI. Concerning proliferation, various conditions increased the median proliferation rate (RPMI, 28%). However, the best conditions were EGF 50 ng/ml (38%), hydrocortisone (44%) and hydrocortisone + EGF 20 ng/ml (50%) (Figure 2A). The Mann–Whitney *U* test only revealed one significant improvement, namely for hydrocortisone + EGF 20 ng/ml (*p* = 0.04) on proliferation.

However, it could be that data averaged over all tumor fragments (Figure 2A), mask a significantly improved viability or proliferation at the individual patient level. Therefore, data were also analyzed per patient (Figure 2B). The absolute (not relative) median difference between the tested conditions and standard RPMI at day 7, of all fragments from one patient, is plotted in Figure 2B. Value ‘0’ stands for no difference between RPMI and the optimization condition. Addition of EGF 50 ng/ml (9%), hydrocortisone (5%) and folic acid 6 mg/L (5%) improved viability when compared to RPMI. Addition of hydrocortisone + EGF 20 ng/ml (5%), folic acid 6 mg/L (4%) and hydrocortisone (3%) improved proliferation of tumor cells in the tissue fragments.

Data from Figure 2A and 2B suggest that optimization conditions containing hydrocortisone, EGF 50 ng/ml or folic acid 6 mg/L supplements would be optimal to improve viability and proliferation of HNSCC cultures. In Figure 2C these three conditions are shown as normalized median values for all fragments per patient at day 7 and related to standard RPMI. Heterogeneity within individual patient biopsies concerning tumor viability and proliferation in response to supplements EGF, hydrocortisone and folic acid, is observed. Also, data suggest EGF 50 ng/ml and folic acid 6 mg/L to be beneficial for individual tumor viability and not proliferation. Hydrocortisone may be beneficial for individual tumor proliferation, however not for tumor viability.

### EGF-Receptor (EGFR)

In head and neck cancer the EGFR is frequently overexpressed [29]. Therefore, we analyzed whether, the supplementation of EGF would increase viability and proliferation of EGFR positive tissue samples. To study this, we cut additional sections from fragments of 7 patients with EGFR positive tumors (green circles, Figure 2C). Five individual patient fragments were cultured with EGF 50 ng/ml (Histocultures 22, 29, 30, 73 and 74) and 1 fragment with hydrocortisone and folic acid 6 mg/L, both to serve as control. We selected 1 to 2 fragments at day 0 and day 7, see Table 4. Surprisingly, addition of EGF at 50 ng/ml did not further improve viability and proliferation of the EGFR positive tumours in the fragments.

**Table 4:** EGF-Receptor expression

Patient	Culture condition	EGFR (%)		Viability (%)	Proliferation (%)
		Day 0	Day 7	Day 7	Day 7
**22**	EGF 50	90	100	30	70
			80	60	50
**24**	Hydrocortisone	80	70	70	10
		40	80	60	25
**26**	Folic acid 6	80	70	30	30
		80			
**29**	EGF 50	50	20	50	30
		40	10	90	2
**30**	EGF 50	90	10	50	0
		90	5	30	30
**73**	EGF 50	100	80	70	10
		90	5	30	0
**74**	EGF 50	95	40	30	30
			50	40	20

Raw percentage of positive EGFR expression at day 0 and day 7.

### Integrity tumor microenvironment during culture

**Figure 3:**
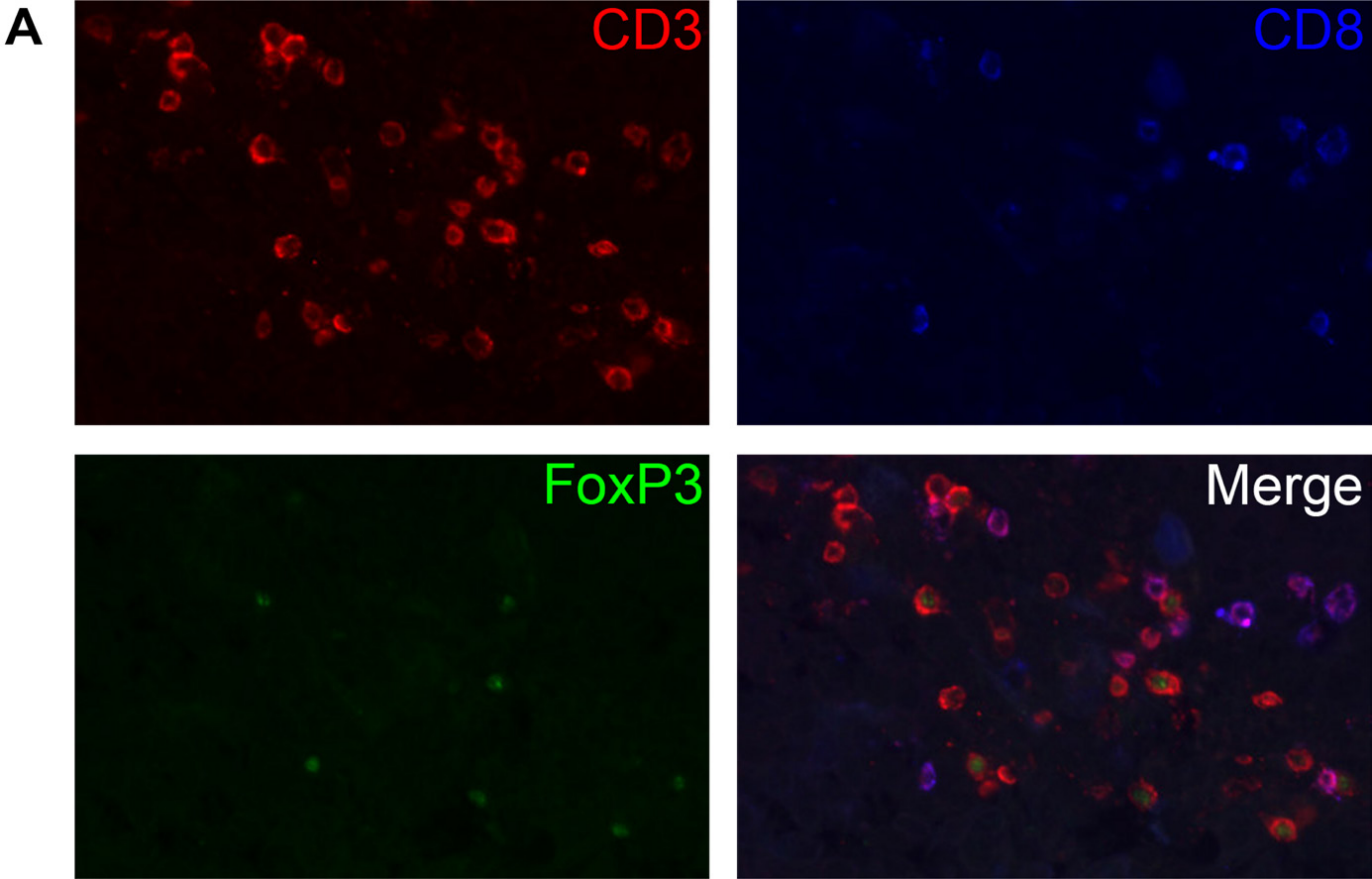

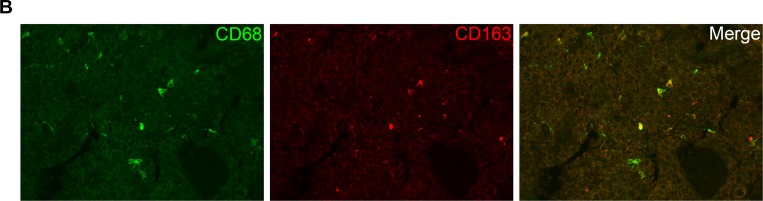
The presence of immune cells during histoculture (**A**) Visualization of CD3, CD8 and FoxP3 T-cell staining at day 7. In the merged image, one can distinguish between T helper cells (CD3+), cytotoxic T cells (CD3+, CD8+) and regulatory T cells (CD3+, FoxP3+). (**B**) Visualization of CD68 and CD163 macrophage staining at day 0. In the merged image, one can distinguish M2 macrophages (CD68/CD163).

To assess the integrity of the tumor microenvironment during histoculture, we performed additional immune stainings on the same sections previously selected for EGFR. One fragment at day 0 and one at day 7 was selected for multiparameter fluorescent immune cell IHC, an example is shown in Figure 3A and 3B. From these immune cell marker stainings, the raw numbers of positive stained immune cell classes per mm^2^ were scored, at day 0 and day 7 (Table 5). Immune cells remained in the tissue of most fragments over the 7-day culture period. There is some variability with some fragments showing a decrease and others showing an increase of immune cell subpopulations when comparing day 0 to day 7 (highlighted cells, Table 5). CD68^+^/CD163^+^ macrophages at day 0 might shift to more CD68^-^/CD163^+^ macrophages at day 7. The number of CD8^+^ cells may decrease during histoculture. There are no significant differences between the total macrophage and T-cell population when comparing day 0 to day 7 (nonparametric unpaired Mann–Whitney *U* test; *p* = 0.073 – 1.000).

**Table 5:** Quantification of immune cell expression

Patient	CD68CD163		CD163		CD68		CD3FoxP3		CD3		CD3CD8		CD3CD8FoxP3	
	Day 0	Day 7	Day 0	Day 7	Day 0	Day 7	Day 0	Day 7	Day 0	Day 7	Day 0	Day 7	Day 0	Day 7
22	3.7	42.5	3.7	10.6	3.7	9.7	38.7	19.4	10.6	7.8	0.0	0.0	0.0	0.0
24	36.7	22.5	2.5	7.0	5.1	9.8	78.8	15.9	36.1	0.0	32.8	0.0	9.9	0.0
26	54.6	20.3	7.1	0.0	2.4	10.1	217.1	41.2	130.3	13.7	111.6	20.6	24.8	0.0
29	0.0	0.0	0.0	0.0	2.2	0.0	6.5	16.1	19.6	32.2	19.6	0.0	0.0	0.0
30	16.7	0.0	0.0	0.0	5.6	11.0	31.1	48.8	56.1	32.5	24.9	16.3	12.5	0.0
73	54.4	58.7	0.0	4.5	38.1	97.9	392.4	222.6	654.1	585.8	697.1	304.6	34.9	11.7
74	33.6	88.7	1.2	7.2	0.0	64.4	6.5	47.7	58.5	160.4	149.6	86.7	0.0	0.0

Values shown are the raw numbers of positive stained immune cells per mm^2^, at day 0 and day 7. The highlighted cells point out an increase of immune cells at day 7 when compared to day 0.

## DISCUSSION

Advanced HNSCC is characterized by an unfavourable 5-year overall survival rate of 35–63% [1–3]. Furthermore, there is a widely divergent individual response to CCRT regimens. Consequently, there is a strong need for a preclinical assay to identify the best treatment for individual patients but also to test novel drugs and drug combinations for these patients.

Since the 1990s various culture models to allow for individualized treatment tests in HNSCC patients, have been published. As HNSCC patients need to start their treatment within 5–6 weeks after diagnosis, *in vitro* screening should be performed preferably within 1–2 weeks to guide decision making. The HDRA assay has led to a culture model most comparable to the *in vivo* tumor with successful culture rates, with a read-out after 7–8 days, and the best correlation between *in vitro* and *in vivo* treatment responses [7]. Despite these promising results, the HDRA is not taken into routine clinical practice, likely for various reasons. First, it is difficult to culture tumor tissue. A laboratory within the hospital is needed in order to quickly transport the fresh biopsies and put them into culture. Secondly, the process of culturing and investigating biopsies is laborious and costly. Thirdly, the specificity and sensitivity of correlating *in vivo* tumor response to *in vitro* HDRA chemosensitivity is relatively low, ranging from 57–78% and 71–91% respectively [18–21]. This could be due the metabolic read-out, used to detect the response of the tumor fragments, as this includes stromal cells and immune cells as well, which might camouflage the specific cancer cell response.

We included immunohistochemistry in our strategy to better determine culture effects on tumor viability and proliferation, and also on the tumor microenvironment which is related to tumor progression, therapy resistance and ultimately patient survival [26, 27]. We also tested various supplements to the standard medium to improve culture conditions.

In our study, 93% of the patient biopsies was successfully cultured. This culture success rate is in agreement with previous literature reporting 88–100% [18, 19, 21, 22]. Hypopharyngeal tumors were more difficult to culture due to a high contamination rate of 13.8% of the fragments. In previous studies [18, 19, 21, 22], the tumor site was never mentioned when patients were excluded due to contamination. We noticed, after microscopic analysis, that more fragments were contaminated with bacteria and fungi than expected. We also noted the culture of benign cells instead of tumor cells, another point not considered in previous studies. Knowing the exact composition of the culture fragments is critical for any conclusion, as illustrated by observations showing that stromal cells are more resistant for cytostatic drugs and radiation *in vitro* [23–25]. When no distinction is made, any chemosensitivity response could represent the response of the tumor microenvironment, benign cells or maybe even contaminations, rather than the tumor cells themselves. By analyzing tumor fragments through immunohistochemistry, we could distinguish tumor cells from benign cells, and also exclude fragments having contaminations. Interestingly, although biopsies were taken from primary tumor sites, 3 patients were excluded since 67% and 100% of the fragments contained mostly benign cells. Furthermore, from the 1451 included tumor fragments we excluded another 104 fragments (7.2%) because of benign tissue presence. The immunohistochemistry read-out also enabled us to see whether a cut-off, arbitrary set to 70% of cancer cells at day 0, would have improved effects on viability and proliferation rates of tumor cells in culture. No significant differences were observed, although tumor fragments containing ≥70% tumor cells at day 0 usually showed higher proliferation rates (*p* = 0.053). There is, however, no evidence that the immunohistochemical read-out provides better correlation with regards to *in vitro* chemosensitivity and clinical response. Nevertheless, immunohistochemical read-out of tumor samples is essential to interpret culture results, as our data show.

Using the standard RPMI medium, the percentage of tumor cells increased from 53% at day 0 to 80% at day 7, while the viability and proliferation of the tumor cells decreased from 90% to 30% and from 30% to 10%, respectively. In order to increase the *in vitro* tumor viability and proliferation at day 7 we tested various optimization conditions. EGF 50 ng/ml, folic acid 6 mg/L and hydrocortisone appeared to improve the viability or proliferation, but there was a patient-to-patient variability between the samples and one condition active for all patients was not identified. We also did not find evidence that EGF is more beneficial in tumor samples overexpressing EGFR. It is unclear whether the EGF recombinant protein is able to penetrate or diffuse efficiently into the tumor tissue when cultured on a sponge.

The wide variety in responses in the culture system could be due to the fact that HNSCCs possess a large degree of intra-tumor genetic heterogeneity [30]. This heterogeneity could lead to a selection bias when culturing HNSCC cells. It is plausible that only the more aggressive subclones stay vital and proliferative during culture. Yet, in a 7-day culture, these subclones will most likely not overgrow the other subclones, only become more dominant [31]. Fact is that the culture does not select for one single subclone –as is the case for organoids and tissue culture cell lines- and therefore can be expected to show a more reliable reflection of the individual patient's tumor. Drug responses in sponge-gel-supported histoculture are therefore expected to better predict drug responses *in vivo*.

There are indeed marked variabilities in the tumor behaviour in the tissue culture system, variabilities that can only be observed by microscopic analysis. In our study, tumor viability and proliferation at day 7 did not significantly relate to stage of disease, tumor site, the abundance of cancer cells at day 0 or the percentage of EGFR expression. However, our comparison of viability and proliferation by stage and tumor was descriptive in nature, rather than testing a specific a priori hypothesis. In this perspective, the *p*-value should be interpreted as indicators of the strength of heterogeneity, given the data we have collected. We interpret the fact that none of the *p*-values are significant as evidence that viability and proliferation are relatively similar within categories of stage and tumor site, given the variability of viability and proliferation within each category. The power of the study did not allow selection of a subgroup of patients with tumor tissue growing more successfully in histoculture. In order to potentially use the histoculture as preclinical individual drug-response assay, a larger window in terms of tumor viability and proliferation at day 7 may be required to assess the efficacy of drugs and/or irradiation. We choose a 7-day read-out in line with earlier HDRA studies in HNC [7], showing good culture success rates and relatively good correlation to the clinic. It could be that shortening of the culture period from 7 to 3–5 days demonstrates higher viability and proliferation rates. On the other hand, cancer cells *in vivo* do not grow at rates as described in cell lines or organoids and have growth rates more similar to those observed in our cultures. But there are still many other variations to test for optimizing the culture system. For example, tumor fragments from one patient (nr 22) were cultured for 5 days. These fragments showed higher rates of viability and proliferation when cultured with EGF. However, reviewing the tumor, it turned out to be an oropharynx tumor with a basaloid SCC histology type, which could be a more rare and aggressive type of cancer [32]. This illustrates that different tumors may have different characteristics in culture, which represents another variable where the pathologist is critical in the assessment of the data.

One obvious advantage of our system over other tumor culture models is that the normal tumor microenvironment is preserved. T-cells and macrophages remain present during 7 days of culture, but again with some variability. Some patients showed a higher number of infiltrating immune cells during culture, and in others the number decreased. Remarkably, in the day 0 fragments almost all macrophages are CD163/CD68 double positive, however after culturing, there is a higher expression of markers for single CD163 and CD68 macrophages. Macrophages exhibiting predominately the anti-inflammatory CD163/CD68 phenotype are known to be tumor-associated M2 macrophages, supporting the tumor, whereas M1 macrophages (CD68) act against the tumor [33]. This switch in phenotype could oppose the grow of tumor cells *in vitro* but this has not been further tested. Although it is an important finding that immune cells are still present after 7 days of culture, we do not have data showing that they are still functionally active or viable. Any such model system limits conclusion on these given the small number of cells and the heterogeneity in the immune cell components observed. Fact is that immune cells do not migrate out of the cultured tissue and be lost for analyses, as our data show. Nevertheless, we have carefully observed the morphology of the macrophages and T-cells infiltrating the tissue before and after culture, and we believe these cells to be still viable as we do not observe any morphological differences: T-cells had a normal rounded shape and FoxP3 expression was very bright in the nucleus. Macrophages had also retained their normal shape and dendrites and had a bright and clear CD68/CD163 staining. So, based on the morphology, we believe that the macrophages were viable, without differences between day 0 and day 7.

In conclusion, the implementation of immunohistochemistry in the sponge-gel-supported histoculture method has provided valuable insights in the quality and interpretation of culturing cancer cells. The histocultures showed decreases in viability and proliferation of tumor cells with marked variation between samples from different patients. This could reflect the natural variability in tumor aggressiveness and tumor type. Our data also show that the tumor microenvironment remains intact although some immune cell types change during the 7-day culture. We report a series of conditions that appear to improve these variations, but a great variability between tumors remains.

In the future, *in vitro* testing of chemotherapeutical agents or irradiation is the next step, preferably in a preclinical setting with tumor tissue from patients derived before treatment. When a good correlation with individual clinical treatment response is found, the histoculture may allow for personalized treatment selection. Also, the assay allows testing of novel treatment agents for this cancer type with a relatively poor prognosis. The heterogeneity of tumors and their microenvironment is preserved which comes closer to reality than cell lines or even organoids. It is in line of these that we expect that our histocultures will allow a better prediction of the optimal treatment for individual HNSCC patients.

## MATERIALS AND METHODS

### Patient selection

The Institutional Review Board of the Netherlands Cancer Institute NKI approved the study and informed consent was obtained from all patients. Tumor samples were obtained from 72 patients with HNSCC in the operating room undergoing either surgery or examination under general anaesthesia, between August 2012 and September 2014. None of the patients received prior chemotherapy or radiotherapy treatment. Only patients with histologically proven squamous cell carcinoma were included.

### Sponge-gel-supported histoculture method

**Figure 4:**
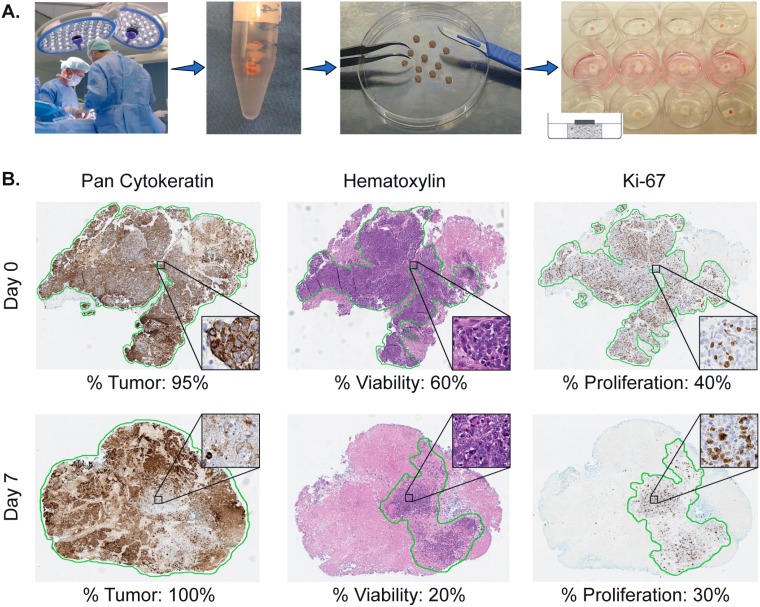
Illustration of the sponge-gel-supported histoculture method and immunohistochemistry read-out (**A**) Biopsies from previously untreated HNSCC patients are taken under general anaesthesia after informed consent. Biopsies are transported to the laboratory within 1 hour and cut into single fragments. Each single fragment is cultured on a sponge drenched into medium in a 12-well plate. The fragment is placed on the sponge in such a way that it is surrounded by air and attached to the sponge enabling it to absorb medium. Using this method, the *in-vivo* situation is simulated. (**B**) Illustration of the pathological scoring system. Of each single tumor fragment, at day 0 and 7, three slides are cut and stained for pan-Cytokeratin, Hematoxylin and Eosin (H&E) and Ki-67. With the H&E and pan-Cytokeratin staining the percentage of tumor is scored (% Tumor, cancer cells). With the H&E staining the percentage of viable cancer cells in relation to the total amount of tumor (including areas of necrosis) is determined (% Viability). The Ki-67 staining is used to determine the proliferation rate of the viable cancer cells (% Proliferation).

The method used in this study, is based on the collagen sponge-gel-supported histoculture utilized before to develop the HDRA, as described by Furukawa in 1995 [16]. Immediately after excision the freshly isolated tumor biopsies were placed in a 15 ml plastic tube containing 10 ml 37° C pre-warmed culture medium (RPMI 1640, Biochrom, cat. no. F1275, without phenol red) supplemented with 10% FBS (Sigma-Aldrich, F7524), L-glutamine (Gibco, 2mM), HEPES (Gibco, 14 mM) and with antibiotics and antimycotics: Amikacin (Sigma-Aldrich, A2324, 20 μg/ml), penicillin and streptomycin (Gibco, 15070, 50 Units/ml and 50 μg/ml), Metronidazole (Sigma-Aldrich, M3761, 25 μg/ml) and Fluconazole (Sigma-Aldrich, F8929, 10 μg/ml). This culture medium was our standard medium, further referred to as ‘RPMI’ in our data. The biopsies were transported to the laboratory within 1 hour after excision. Subsequently, the tumor tissue was placed on a Petri Dish (BD Falcon, 100 × 20 mm) and rinsed twice with PBS to minimize microbial contamination. Next, the biopsies were mechanically minced with scalpels into 1–2 mm^3^ fragments. From each biopsy, three to six fragments were immediately fixated in 4% formalin to determine ‘day 0’ control values. The remaining fragments were each placed on individual sponges (Pfizer, Gelfoam absorbable gelatin sponge, 12–7 mm, Brocacef, cut into 0.5 cm squares), which were first placed into individual wells (BD Falcon, 12-well Multiwell plate) with 1 ml medium and cultured at 37° C in 5% CO_2_ atmosphere. Three to six fragments were cultured in the above-mentioned standard ‘RPMI’ control medium, the remaining fragments were used to test various culture conditions. A simplification of the culture method is shown in Figure 4A. The cultured fragments were harvested after completion of the 7-day culture period. These fragments were removed from the sponges with forceps and every single fragment was placed into an individual biopsy cassette (Klinipath), which was then immediately transported into a 4% formalin fixation solution (Klinipath, 4090-9010, diluted with demi-water) for at least 24 hours.

### Optimization conditions

A variety of conditions were tested aiming to potentially improve the above-mentioned standard ‘RPMI’ culture condition. ‘RPMI’ was compared to the following optimization conditions: Hydrocortisone supplement (Sigma, H4001): 0.4 μg/ml; Epidermal growth factor supplement (EGF, PeproTech, AF-100-15): 5 ng/ml, 20 ng/ml and 50 ng/ml; Folic acid supplement (Sigma-Aldrich, F8758): 6 mg/L, 11 mg/L and 21 mg/L; DMEM/F-12 + GlutaMax medium (Gibco, 31331) with all the supplements of our standard RPMI medium; and refresh medium every 2 days. All conditions were tested in tissue samples derived from at least 10 patients (involving 30 to 60 tumor fragments).

### Immunohistochemical analysis

After formalin fixation, the samples were processed via a Tissue Processor machine (Excelsior, Thermo Scientific. Reagents: formaldehyde, alcohol, xylene and paraffin) and thereafter embedded in paraffin, sectioned and placed onto slides. Immunohistochemistry was performed on the BenchMark Ultra automated staining instrument (Ventana Medical Systems). Paraffin sections were cut at 3 μm and heated at 75° C for 28 minutes and deparaffinized in the instrument with EZ prep solution. Sections were treated with Cell Conditioning 1 buffer (CC1, Ventana Medical Systems) for 36 minutes (Ki-67, pan-Cytokeratin) or 64 minutes (EGFR) at 95° C before incubation with the primary antibodies (Ventana Medical Systems).

To analyze tumor characteristics we used the standard Hematoxylin and Eosin (H&E) staining and the immunohistochemical stainings Ki-67 (nuclear staining) and pan-Cytokeratin (cytoplasmic staining). For the Ki-67 staining, sections were incubated in a 1:250 dilution of the primary antibody (clone MIB-1, DAKO) for 32 minutes at RT. For the pan-Cytokeratin staining, sections were incubated in a 1:100 dilution of the primary antibody (clone AE1/AE3, Thermo Scientific) for 32 minutes at RT. Bound primary antibody was detected using the Universal DAB Detection Kit (Ventana Medical Systems) and slides were counterstained with Hematoxylin. To investigate tumor infiltrating immune cells we used multiplex fluorescent immunohistochemistry stained slides for CD4+ helper T-cells (CD3+, CD8-, FoxP3-), regulatory T-cells (CD3+, FoxP3+), CD8+ cytotoxic T-cells (CD3+, CD8+) and CD8+ regulatory T-cells (CD3+, CD8+, FoxP3+). We also stained for M1 macrophages (CD68^+^) and M2 macrophages (CD68^+^/CD163^+^). Two different primary antibody combinations were used for overnight incubation: CD3 (1:100, ab828 rabbit polyclonal antibody; Abcam, Cambridge, UK), CD8 (1:100, mouse monoclonal IgG2b, 4B11; Novocastra, Newcastle-upon-Tyne, UK), FoxP3 (1:100, mouse monoclonal IgG1, clone 236A/E7; Abcam) and CD163 (clone 10D6, Novocastra NCL-CD163) / CD68 (clone 514H12; ab49777; Abcam). Alexa Fluor labeled Goat-anti-rabbit-A546 (red), Goat-anti-mouse-IgG2b-A647 (blue) and Goat-anti-mouse-IgG1-A488 (green) (Invitrogen, Life Technologies, Carlsbad, USA) were used for visualizing the T-cell markers. Alexa Fluor labelled Goat-anti-mouse-IgG2a-A488 and Goat-anti-mouse-IgG1-A546 (Invitrogen-Molecular Probes, Eugene, OR) were used for CD68 and CD163 detection. Slides were mounted using VectaShield mounting medium containing DAPI (Vector Laboratories, Burlingame, USA). Immunofluorescent images were acquired with an LSM700 confocal laser scanning microscope equipped with an LCI Plan-Neofluar 25x/0.8 Imm Korr DIC M27 objective (Zeiss, Göttingen, Germany) and analyzed with the LSM software.

EGF-Receptor (EGFR) was detected using clone 5B7 (ready-to-use dispenser, 16 minutes at 37° C, Roche). Bound antibody was detected using the UltraView DAB Detection Kit (Ventana Medical Systems). Slides were counterstained with Hematoxylin II and Bluing Reagent (Ventana Medical Systems).

At our institute, HPV status of oropharyngeal tumors is determined with the surrogate IHC markers p53 and p16^ink4a^ as described in literature [34].

### Pathologist scoring read-out

The immunohistochemistry slides were analyzed by experienced research pathologists (JS, EJ), blinded for the conditions. In order to make a reliable calculation we took a cut-off of at least 3 available fragments for the day 0 control, the standard ‘RPMI’ day 7 control and for the various tested conditions. Consequently, for each biopsy, three to six fragments were used to determine ‘day 0’ values, and three to six fragments were used at day 7 per culture condition. Per cultured tumor fragment three histological slides were cut, stained and scored for percentage of tumor, viability and proliferation. The percentage of tumor (abundance of cancer cells in the tissue fragment) was assessed using the H&E staining. This was subsequently verified with the pan-Cytokeratin staining (see % Tumor, Figure 4B). Of note, while scoring for % Tumor, stromal tissue and infiltrate were always excluded. The pathologist also scored for tumor viability using the H&E slides (see % Viability, Figure 4B) by estimating the percentage of viable cancer cells within the total amount of cancer cells. Cells were scored viable when specific signs and characteristics of cell death (like pyknosis, karyorrhexis, karyolysis and eventually disappearance of the cell nucleus) were absent. The percentage of viability assessed, is solely the viability of the total number of all cancer cells, while excluding again stromal tissue and infiltrate.

Ki-67 was used to determine the proliferation rate of the viable cancer cells (see % Proliferation Figure 4B). Therefore, for each successfully cultured fragment, three percentages were scored. An example of our scoring system is shown in Figure 4B.

The EGFR expression was scored as percentage of positive tumor cells. The T-cell and macrophage subpopulations, as determined by multiparameter fluorescent IHC, were scored as number of cells per mm^2^ in the total tumor section.

### Analyses and statistics

The scoring results are presented in this manuscript as either raw or normalized data. Raw data were used to present the actual success of the histoculture technique. Normalized data were used for the analyses between patients. To normalize the data per patient, median percentages (tumor, viability and proliferation) at day 0 were calculated. Consequently, all single fragment scoring percentages at day 0 and day 7, were normalized against this median value at day 0. These data will further be referred to as the ‘normalized data’. This analysis method was done in order to deal with the tissue heterogeneity issues that exist within HNSCC and therefore this method corrects for the variability between the fragments at day 0. Beside this, we were now also capable of comparing data between patients.

To see whether we were able to optimize our standard ‘RPMI’ medium by adding various supplements, the ‘RPMI’ condition served as the control condition to which the optimization conditions were compared. This was done by comparing the normalized median percentage of each condition, which was calculated as the median percentage of all normalized percentages per tested condition.

Descriptive statistics were gathered using GraphPad Prism 4.0b. Data were analyzed using IBM SPSS Statistics 23.0. Figure 2C was conducted using R version 3.1.3. Overall, *p* values < 0.05 were considered significant.
